# Complete Plastid Genome of the Brown Alga *Costaria costata* (Laminariales, Phaeophyceae)

**DOI:** 10.1371/journal.pone.0140144

**Published:** 2015-10-07

**Authors:** Lei Zhang, Xumin Wang, Tao Liu, Haiyang Wang, Guoliang Wang, Shan Chi, Cui Liu

**Affiliations:** 1 Laboratory of Genetics and Breeding of Marine Organism, College of Marine Life Sciences, Ocean University of China, Qingdao, People’s Republic of China; 2 CAS Key Laboratory of Genome Sciences and Information, Beijing Key Laboratory of Genome and Precision Medicine Technologies, Beijing Institute of Genomics, Chinese Academy of Sciences, Beijing, People’s Republic of China; Laboratoire Arago, FRANCE

## Abstract

*Costaria costata* is a commercially and industrially important brown alga. In this study, we used next-generation sequencing to determine the complete plastid genome of *C*. *costata*. The genome consists of a 129,947 bp circular DNA molecule with an A+T content of 69.13% encoding a standard set of six ribosomal RNA genes, 27 transfer RNA genes, and 137 protein-coding genes with two conserved open reading frames (ORFs). The overall genome structure of *C*. *costata* is nearly the same as those of *Saccharina japonica* and *Undaria pinnatifida*. The plastid genomes of these three algal species retain a strong conservation of the GTG start codon while infrequently using TGA as a stop codon. In this regard, they differ substantially from the plastid genomes of *Ectocarpus siliculosus* and *Fucus vesiculosus*. Analysis of the nucleic acid substitution rates of the Laminariales plastid genes revealed that the *petF* gene has the highest substitution rate and the *petN* gene contains no substitution over its complete length. The variation in plastid genes between *C*. *costata* and *S*. *japonica* is lower than that between *C*. *costata* and *U*. *pinnatifida* as well as that between *U*. *pinnatifida* and *S*. *japonica*. Phylogenetic analyses demonstrated that *C*. *costata* and *U*. *pinnatifida* have a closer genetic relationship. We also identified two gene length mutations caused by the insertion or deletion of repeated sequences, which suggest a mechanism of gene length mutation that may be one of the key explanations for the genetic variation in plastid genomes.

## Introduction

*Costaria costata* is an annual marine alga that belongs to the class Phaeophyceae, order Laminariales, and family Costariaceae. It grows naturally along the northern coast of the Pacific Ocean. In Asia, it is mainly distributed in the coastal waters of Japan and the Korean Peninsula [1], and has been newly cultivated in Korea [2]. *C*. *costata* propagates rapidly and therefore plays a central role in the restoration of marine pastures and construction of sea forests. In addition to its dietary value for marine animals such as abalone and sea urchins, *C*. *costata* is also important for the seaweed industry because it is used in the extraction of alginate, mannitol, and seaweed starch [3, 4]. *C*. *costata* is not distributed in China. However, because it has significant economic value and can potentially be cultivated, it was introduced in China in 1992. Sinc then, experiments have been carried out to optimize indoor cultivation [5]. In 2007, *C*. *costata* was successfully reproduced and bred in the sea areas of Dalian in the Liaoning Province. Currently, small-scale farming of *C*. *costata* is carried out in the sea areas of both Dalian and Rongcheng in North China.

The study of *C*. *costata* gradually intensified in recent years, mainly because this alga contains unique and abundant biologically active substances and sulfated fucan [6]. Previous research has shown that its dietary fiber has significant hypolipidemic effects [7]. However, with the exception of species taxonomy, population structure analysis, and its mitochondrial genome [8–11], little information about the molecular biology of *C*. *costata* has become available to date. Prior research has been restricted mainly to methods that use normal molecular markers, such as restriction fragment length polymorphism and small-subunit ribosomal DNA.

Plastids are important photosynthetic organelles with their own genomes. Plastid DNA is compact and has a fast evolutionary rate, which make it an ideal tool for evolutionary and population studies. The *rbc*L, *matK*, and *psbA* plastid genes are good taxonomic barcodes that are often used for species identification [12]. In recent years, determination of the evolution rates of plastid genomes has become an active area of research [13]. Rates of nucleotide substitution provide appropriate windows of resolution for the study of plant phylogeny at deep evolutionary levels. A description of the molecular change based on the complete plastid genome is essential for a full understanding of the mechanisms of mutation and evolutionary change [14]. Plastids arose through a process of primary and secondary endosymbiosis [15]; thus, unraveling their origin and evolution has been a challenging scientific puzzle. Phylogenetic analysis at the whole plastid genome level provides a more comprehensive and accurate mean to clarify the evolution of plastids.

We determined the complete plastid genome sequence of the large brown alga *C*. *costata* with next-generation sequencing. The sequence represents the first fully characterized plastid genome from the newly identified family of Costariaceae. In addition, we explored the evolutionary position of this alga based on the plastid genomic data currently available for other algae and higher plants. *Saccharina japonica* and *Undaria pinnatifida* have been main cultivated large brown seaweeds in China for decades. *C*. *costata* has great potential to follow these species in large-scale farming. All three algae belong to the order Laminariales. The present study not only contributes to efforts to exploit the advantageous characteristics of brown algae but also provides a scientific basis for the development of new algal breeds and further exploration of new marine sugar resources.

## Materials and Methods

### DNA Extraction

Algal material was provided by the Culture Collection of Seaweed at Ocean University of China in Qingdao (sample number: 2012050110). Gametophytes of *C*. *costata* were cultivated at 8–12°C in sterilized filtered seawater under fluorescent light (3000 lux; 12 h light/dark cycles). The gametophytes were concentrated on filter paper and subsequently washed three times with sterilized filtered seawater. Total DNA was extracted from fresh drained material according to a modified cetyltrimethylammonium bromide method [16].

### Genome Sequencing and Assembly

Approximately 5 μg of purified DNA was used for the construction of short-insert libraries following the manufacturer’s protocol (Illumina Inc., San Diego, CA, USA). The sequenced libraries included short pair-end libraries with insert lengths of 250 bp, 300 bp, and 500 bp. Library construction and sequencing were performed by the Beijing Genomics Institute (Shenzhen, China).

The raw sequence reads corresponding to the plastid DNA were detected based on their similarity to the plastid genomes of *U*. *pinnatifida*, *S*. *japonica*, *Ectocarpus siliculosus*, and *Fucus vesiculosus* by using the Basic Local Alignment Search Tool (BLAST) software [17, 18]. For the short insert libraries, a total of 2,096,479 raw reads corresponding to plastid DNA were obtained with an average read length of 101 bp. The raw data provided approximately 1600-fold coverage of the plastid genome. Subsequently, these plastid-related reads were assembled by using SOAPdenovo software [19] with default assembly parameters (SOAPdenovo all -s assembly.conf -o mysample -K 49 -p 8 &). All the assembled contigs were aligned and ordered with respect to the reference plastid genomes using MEGA 6.0 software [20]. The sequence of the circular genome was completed via manual assembly. Polymerase chain reaction amplification and Sanger sequencing with the primers listed in S1 Table were performed to fill in the gaps and confirm the four junction regions.

### Annotation and Analyses

Protein-coding genes and open reading frames (ORFs) were identified with BLASTN and BLASTX searches of the National Center for Biotechnology Information database [21]. The ribosomal RNA (rRNA) genes were identified via sequence alignment with known plastid genes of *S*. *japonica* and *U*. *pinnatifida*. The transfer RNA (tRNA) genes were identified by using tRNAscan-SE1.21 software [22]. The physical map of the circular genome was drawn with OGDRAW software [23]. Sequence alignment was performed and base composition determined by using BioEdit software. Gene substitution rates were calculated with MEGA 6.0 software [20]. Co-linear analyses of the five plastid genomes were conducted with Geneious software (version R7 7.0.6) [24].

### Phylogenetic Analysis

Phylogenetic analysis was performed with plastid genomes from 43 taxa, including green algae, red algae, and brown algae, as well as two land plants. *Cyanophora paradoxa* was assigned as an outgroup. A list of 22 shared plastid genes were used for phylogenetic analysis (*atpA*, *atpB*, *atpH*, *rbc*L, *petB*, *petG*, *psaA*, *psaB*, *psaC*, *psbA*, *psbB*, *psbC*, *psbD*, *psbE*, *psbF*, *psbL*, *psbN*, *rpl20*, *rps11*, *rps12*, *rps14*, and *rps19*). Sequences were aligned with MEGA 6.0 software and edited manually. MrBayes v3.1.2 software [25] was used to investigate the evolutionary relationships based on 6,104 amino acids corresponding to the 22-protein dataset. Bayesian inference analysis was performed with two separate sequence analyses for four Markov chains (using default heating values), which were run for 500,000 generations, discarding the first 25% as burn-in [26]. The remaining trees were used to build a 50% majority rule consensus tree, accompanied by posterior probability values. FigTree v1.3.1 (http://tree.bio.ed.ac.uk/) was used to generate the phylogenetic trees.

## Results and Discussion

### Genome Organization and Comparison

**Fig 1:**
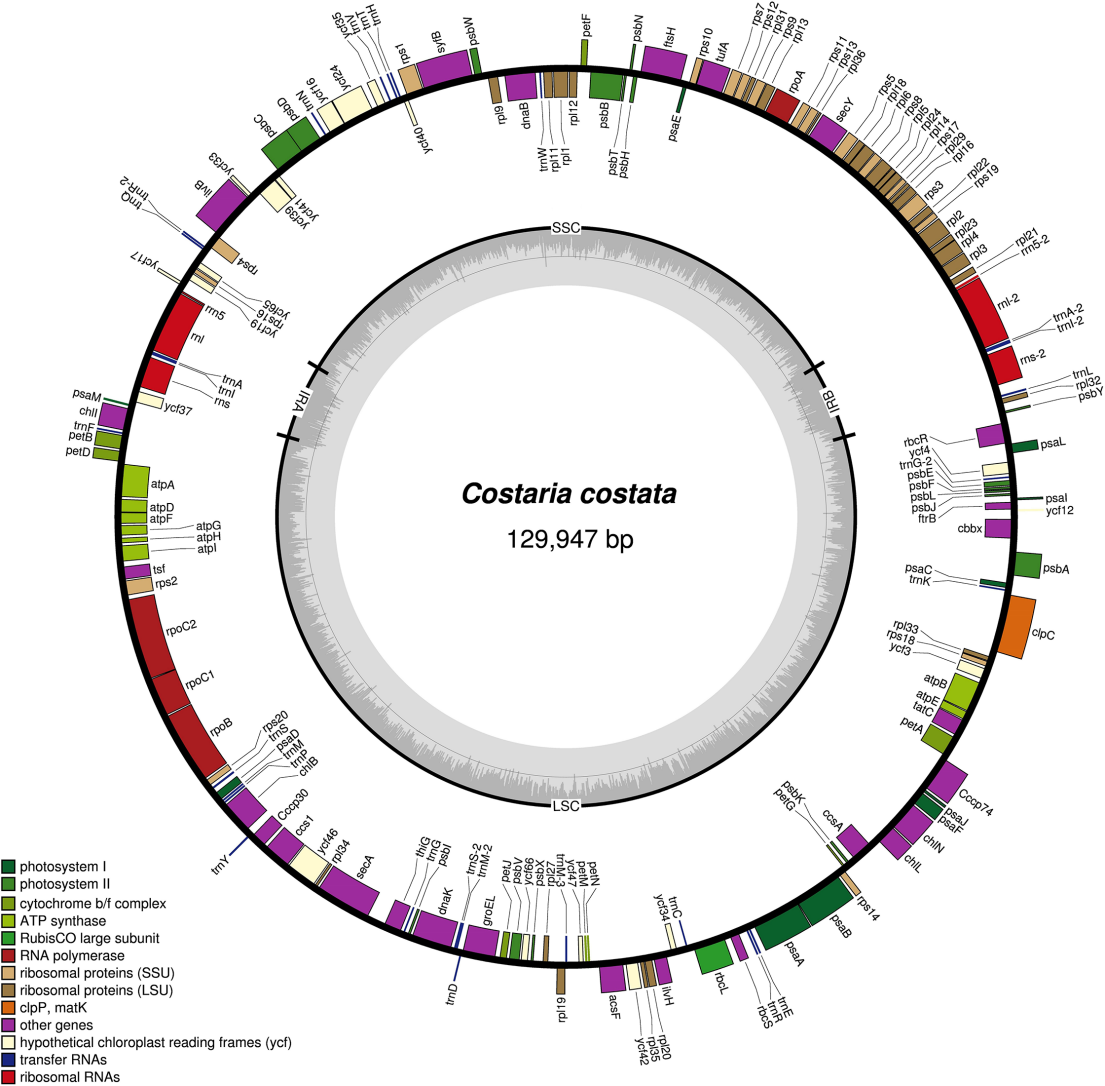
Gene map of the *Costaria costata* plastid genome. Genes on the outside of the map are transcribed counterclockwise and those inside the map are transcribed clockwise. The innermost ring in gray represent the GC content.

The plastid DNA of *C*. *costata* is a 129,947 bp circular molecule. It contains a large single-copy (76,507 bp) and a small single-copy (42,622 bp) region separated by two inverted repeats (IRa and IRb: 5,409 bp), as shown in Fig 1. The overall A+T content is 69.13%, which is the second-lowest among the published Phaeophyceae plastid genomes to date (Table 1). The *C*. *costata* plastid genome contains 139 protein-coding genes including two ORFs, 27 tRNA genes, and six ribosomal RNA genes. None of the genes contains introns. The gene content and order are almost identical to those of the plastid genomes of *S*. *japonica* and *U*. *pinnatifida*; however, *C*. *costata* encodes fewer tRNAs. Similar to other reported species, the plastid genome of *C*. *costata* is compact, with an intergenic region that accounts for approximately 17.31% of the entire genome. Four pairs of genes overlap over 4 to 13 nucleotides.

**Table 1:** General features of five brown seaweed plastid genomes.

Species	Size (bp)	A+T (%)	IR (bp)	Genes	tRNAs
*Fucus vesiculosus*	124986	71.05	4863	139	26
*Ectocarpus siliculosus*	139954	69.33	8615	148	27
*Saccharina japonica*	130584	68.95	5496	139	29
*Undaria pinnatifida*	130383	69.38	5404	139	28
*Costaria costata*	129947	69.13	5409	139	27

In a comparison of the five known brown algal plastid genomes from Phaeophyceae, co-linear analysis showed that the protein-coding gene content and order in all three species from the Laminariales order are virtually identical (S1 Fig). All three have inverted repeats (IR) regions of similar length. Comparisons of plastid genome structures at the order level found significant differences between Laminariales, Fucales, and Ectocarpales but no apparent pattern in the overall genome structure. However, two long and conservative gene clusters with lengths of 30.7 Kb and 34.9 Kb containing 49 and 37 genes, respectively, were found among the five plastid genomes analyzed. In these gene clusters, the gene order of all five species is exactly the same, which demonstrates a conserved co-linear relationship between brown algal plastid genomes.

### Codon Usage

The plastid genome of *C*. *costata* has three types of start codons. Whereas ATG is used for nearly all of the plastid genes, *rps8* and *psbF* have GTG as the start codon. Remarkably, the same is true for *U*. *pinnatifida* and *S*. *japonica* (Table 2). In addition, ATT is the start codon for the *atpA* gene in *C*. *costata* and *U*. *pinnatifida* but not that in *S*. *japonica*. Three typical stop codons (TAA, TAG, and TGA) are found in the plastid genes of *C*. *costata* with preferences of 82.73% for TAA and 14.39% for TAG. The TGA stop codon is used only in the *ycf24* gene, which also has a TGA stop codon in *S*. *japonica* and *U*. *pinnatifida*. The *ycf40* gene in the *U*. *pinnatifida* plastid genome, also has a TGA stop codon.

**Table 2:** Comparison of codon use in five large brown algal plastid genomes.

		Start codon			Stop codon		
Order	Species	ATG	GTG	ATT	TAA	TAG	TGA
Fucales	*F*. *vesiculosus*	136	2 (*rpl3*, *psbF*)	1 (ORF76)	113	17	9 (*ycf24*, *chlI*, *thiG*, *ycf54*, ORF501, *rbcR*, *rps10*, *rps9*, *rpl29*)
Ectocarpales	*E*. *siliculosus*	145	3 (*rpl3*, *rps8*, *rbcR*)	0	129	14	5 (*ycf24*, *ycf19*, *rps1*, *rps7*, *rpl31*)
Laminariales	*S*. *japonica*	137	2 (*rps8*, *psbF*)	0	114	24	1 (*ycf24*)
	*U*. *pinnatifida*	136	2 (*rps8*, *psbF*)	1 (*atpA*)	116	21	2 (*ycf24*, *ycf40*)
	*C*. *costata*	136	2 (*rps8*, *psbF*)	1 (*atpA*)	118	20	1 (*ycf24*)

Numbers in the table represent the plastid gene codon usage in 5 species.

ATG is the most commonly used translation initiation codon in all species studied thus far. Besides ATG, GTG serves as initiation codon in some bacterial and several red algal plastid genes [27]. Several examples exist in which the ATG start codon has mutated to ATT, which in turn stabilizes the binding of the initiator tRNA^Met^ to the ribosome [28]. The plastid genomes of *E*. *siliculosus* and *F*. *vesiculosus* contain three and two genes, respectively, that have GTG start codon. In the five plastid genomes from Phaeophyceae, GTG start codon usage is concentrated in four genes (*rpl3*, *rps8*, *rbcR*, and *psbF*). The three plastid genomes from the same order of Laminariales show the same pattern of GTG start codon usage (see Table 2). Thus, the use of GTG as a start codon in these genes is strongly conserved.

**Fig 2:**
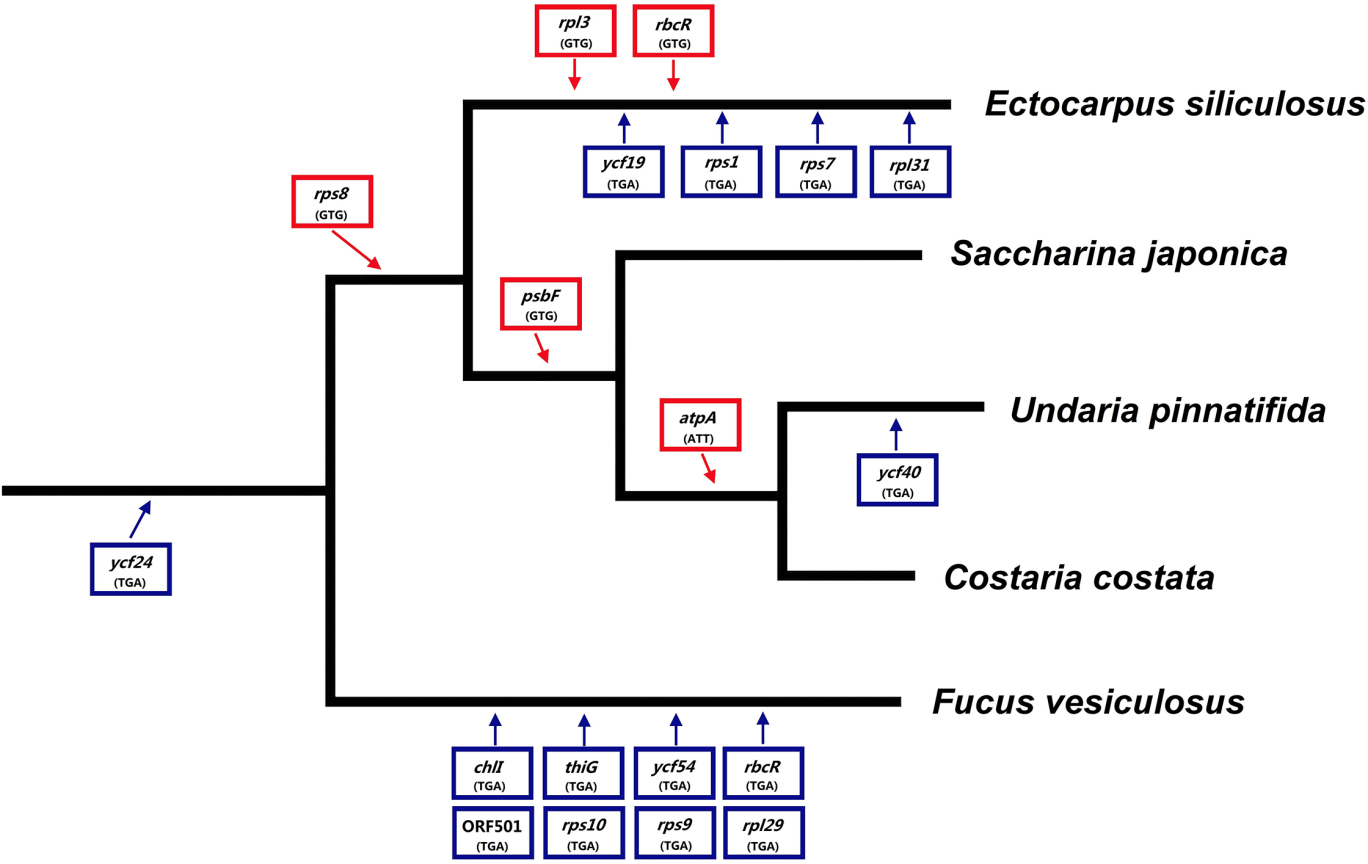
Evolutionary patterns of the start and stop codons of the plastid genes of five Phaeophyceae species. Red boxes above the line represent the genes and their start codons; blue boxes below the line represent the genes and their stop codons.

TAG is the second most commonly used stop codon, after TAA. The frequency of the TAG stop codon in the plastid genes of five species from Phaeophyceae is 9.46–17.27%. As reported previously, in some cases, especially in the mitochondrial genome, TGA encodes tryptophan instead of a stop codon [29]. In plastid genes, however, TGA is a normal stop codon, and in *E*. *siliculosus* and *F*. *vesiculosus* we identified five and nine genes, respectively, with TGA stop codons. By contrast, in the three species of Laminariales, only one or two genes have TGA stop codons (*ycf24*, *ycf40*). Thus, Laminariales species use few TGA stop codons, differing substantially from *E*. *siliculosus* and *F*. *vesiculosus* plastid genomes. Based on the differences in the start and stop codons in plastid genes, as well as the evolutionary relationships between the five species from Phaeophyceae, we inferred a possible evolutionary pattern for their plastid genes (Fig 2).

### Gene Substitution Rates

We calculated and compared the gene substitution rates of all the three plastid genomes of Laminariales, and the results demonstrated that the nucleotide substitution rates of all 137 protein-coding genes ranges from 0 to 20.88% with an overall substitution rate of 9.55%. The *petF* gene has the highest substitution rate (20.88%), and the *petN* gene has the lowest substitution rate (0; S2 Table). Analysis of the amino acid substitution rates showed an overall substitution rate of 5.57% for all genes, which is lower than the rates at the nucleotide level and indicates a certain proportion of synonymous substitutions. The overall comparison between different Laminariales species showed that plastid gene variation between *C*. *costata* and *S*. *japonica* is lower than that between each of those species and *U*. *pinnatifida*. The results showed that *C*. *costata* and *S*. *japonica* have a closer relationship, which differs from the results of the phylogenetic relationship analysis.

The mitochondrial genomes of the three Laminariales species *C*. *costata*, *S*. *japonica*, and *U*. *pinnatifida* have been published [30–32]. Therefore, we also calculated and analyzed the gene substitution rates among them. The results showed that the mitochondrial gene substitution rates ranges from 7.46% to 28.20% at the nucleotide level. The *rps11* gene has the highest substitution rate and the *atp9* gene has the lowest substitution rate (S3 Table). The overall comparative analysis of the three mitochondrial genomes indicated that the lowest interspecies substitution rates is between *S*. *japonica* and *C*. *costata*, which is in line with the results of the plastid gene analysis. Analyses of both plastid and mitochondrial genes support a closer phylogenetic relationship between *S*. *japonica* and *C*. *costata* than between each of these species and *U*. *pinnatifida*. Despite the plastid genome having a conservative rate of evolution and stable gene content, comparative molecular analyses revealed complex patterns of mutational change. Previous research found the silent site substitution rate of the mitochondrion to be lower than those of the plastid and nucleus in land plants [33]. However, recent organelle genome analyses from lineages outside of land plants suggest the opposite, that the substitution rate of plastids is lower than that of their mitochondria [13]. The three species used in our research belong to Laminariales and are related close enough for substitution rate analyses. Our results support that the plastid substitution rate is lower than that of the mitochondrion in algae groups with primary or secondary plastids.

### Gene Length Change

**Fig 3:**
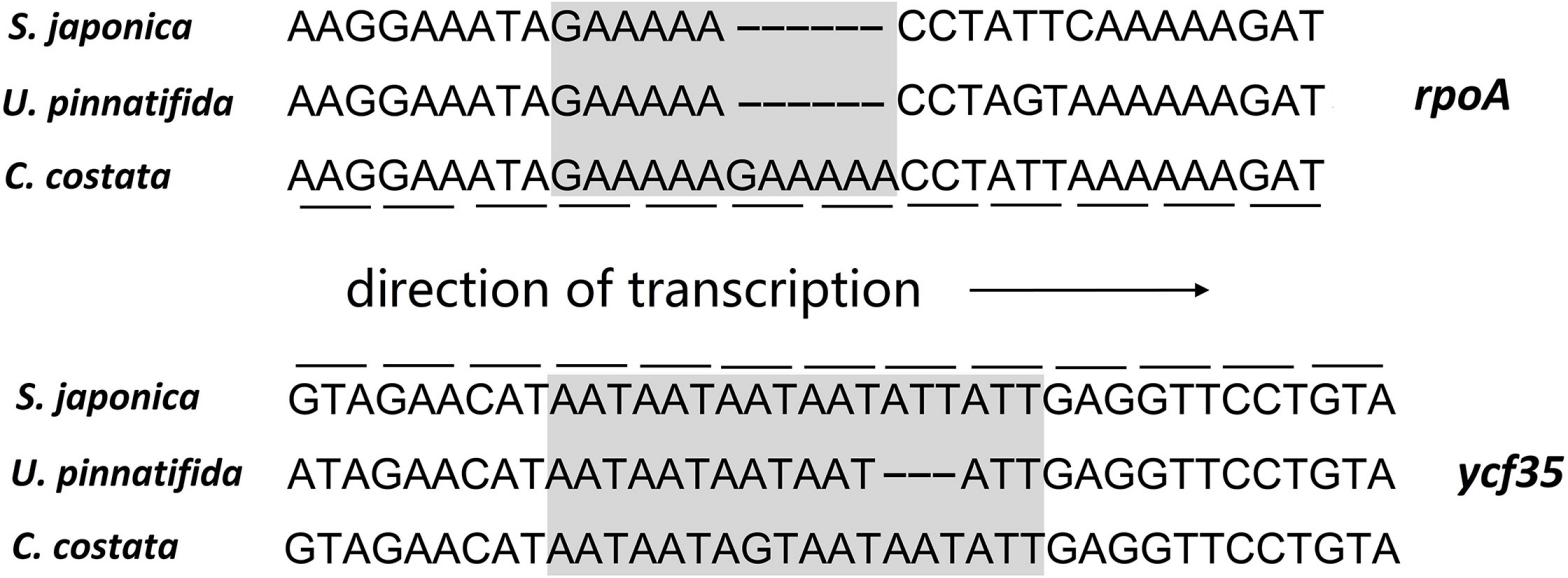
The 3-bp and 6-bp length mutations in *C*. *costata* plastid genes. The lines correspond to the amino acid triplet codon of each gene for three species, and the dash (–) represents the deletion of a base.

The results of a comparison of the genetic differences between three Laminariales plastid genomes demonstrated differences in *ycf35* and *rpoA* gene length between species. These gene length differences are caused by simple sequence repeats or deletions. The *rpoA* gene in the *C*. *costata* plastid genome is 6 bp longer than those in *S*. *japonica* and *U*. *pinnatifida* owing to a GAAAAA simple sequence repeat. However, neither the ORF nor the overall structure of the gene was affected by this 6 bp duplication. It may have formed during the process of gene replication, and we can infer that the duplication occurred after the differentiation of the families Costariaceae and Alariaceae. For the *ycf35* gene, an insertion or deletion of a simple sequence of ATT or AAT makes its gene length 3 bp shorter in *U*. *pinnatifida* than those in *S*. *japonica* and *C*. *costata* (Fig 3). Neither the 3-bp nor the 6-bp length change influences the corresponding gene frame, but the mutations result in differences in both gene length and sequence.

Simple sequence repeats, also called microsatellites, are an important source of gene recombination and variation [34]. In this study, we identified two gene length changes caused by insertion or deletion of a 3-bp or 6-bp repeat sequence in two plastid genes. These changes do not alter the original gene reading frame. Previous studies have shown that duplication or deletion of short repeated sequences are usually generated during the genome replication process, which cause gene length mutation [35]. All results and analyses of gene length changes in this study were verified and validated by polymerase chain reaction amplification and Sanger sequencing. Our results revealed a mechanism of gene length mutation that may be one of the most important sources of genetic variation in the plastid genome.

### Phylogenetic Analyses

**Fig 4:**
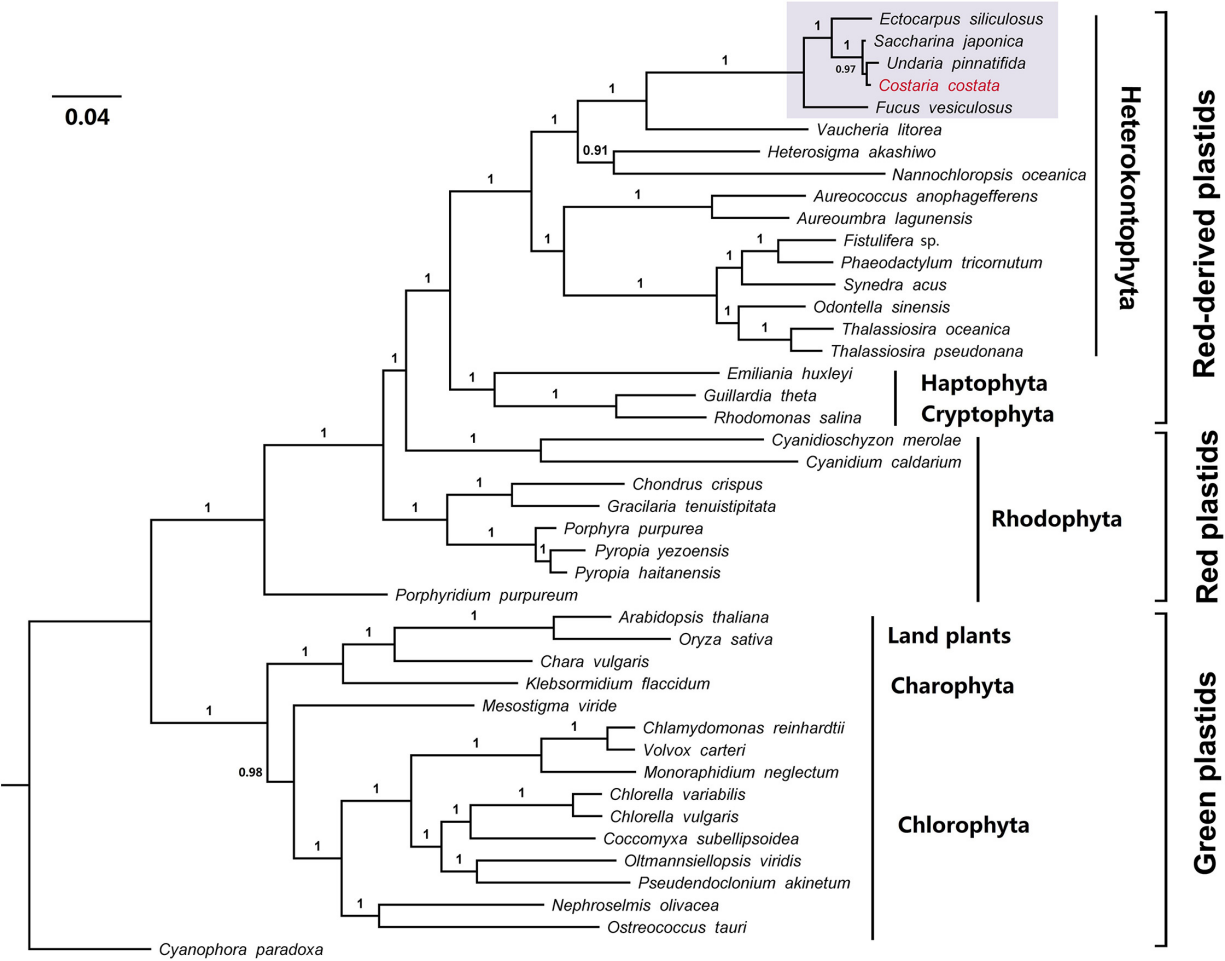
Phylogenetic tree (Bayesian inference) of 43 taxa based on 22 plastid protein-coding genes.

The phylogenetic tree with posterior probabilities based on 22 plastid protein-coding genes is presented in Fig 4. All taxa are clearly separated into two groups; one group consists of the green algae, Charophyta and land plants and represents the green plastid lineage branch. The other group represents the red plastid lineage that includes *Porphyridium purpureum* (emerging at the base of this branch), as well as red plastids and red-derived plastids. The red-derived plastid subgroup includes Haptophyta, Cryptophyta, and Heterokontophyta. Among the sub-branches of five brown algae belonging to the class Phaeophyceae, *F*. *vesiculosus* is the most isolated branch, followed by *E*. *siliculosus*. All three species from the order Laminariales form a clade, in which *C*. *costata* and *U*. *pinnatifida* receive a relatively high posterior probability that support a close relationship and are further grouped with *S*. *japonica*. This result does not correspond to the results of the gene substitution rates analysis performed in this study, and this inconsistency may be caused by the use of different data including only 22 protein-coding genes. In summary, all of our phylogenetic analyses support the conclusion that “primary” plastids are of both red and green lineage [36]. Nevertheless, in the red plastid lineage, *P*. *purpureum* was designated the most isolated branch and shows only a limited genetic relationship with other species. Considering that this plastid is a unicellular marine red alga and its gene order differs from that of other Rhodophyta, *P*. *purpureum* may represent a novel evolutionary plastid group [37].

In China, *S*. *japonica* and *U*. *pinnatifida* are currently the most important cultivated brown seaweeds farmed on a large scale. However, the use of single aquaculture species has seriously limited the development of the Chinese seaweed industry. Decades of simple and single breeding methods have gradually reduced the biodiversity of coastal aquaculture waters, and it is challenging to use cultivated seaweeds in the restoration of coastal ecosystems. *C*. *costata* is mainly distributed in the colder waters of the North Pacific, and normally grows attached to rocks. According to the results of this study, *C*. *costata* is closely related to *S*. *japonica* and *U*. *pinnatifida* phylogenetically. Therefore, it is almost impossible for *C*. *costata* to grow wildly in the temperate waters in China or damage the oceanic ecological balance and marine environment. Similar to *S*. *japonica* when first introduced in China, *C*. *costata* is a new type of large brown seaweed that has now been proven to be able to grow and reproduce in Dalian sea areas. It is urgent to accelerate the scientific study of the breeding and large-scale farming of this new species, which will be of great importance in the further development of the seaweed industry in China.

## Supporting Information

S1 Fig Comparison of five large brown algal plastid genomes from Phaeophyceae with Geneious software. The shadow indicates the two conserved gene clusters.
(TIF)

S1 Table Sequences of the primers designed for gap filling and assembly validation. (PDF)

S2 Table Substitution rates of 137 shared protein-coding genes among three plastid genomes from Laminariales. (PDF)

S3 Table Substitution rates of 35 shared protein-coding genes among three mitochondrial genomes from Laminariales. (PDF)

## References

[1] Selivanova ON, Zhigadlova GG, Hansen GI. Revision of the systematics of algae in the order Laminariales (Phaeophyta) from the Far-Eastern seas of Russia on the basis of molecular-phylogenetic data. Russ J Mar Biol. 2007; 33: 278–289.

[2] Sohn CH. Developments on two newly cultivated species *Capsosiphon fulvescens* and *Costaria Costata* in Korea. J Phycol. 2003; 53–54.

[3] Imbs TI, Shevchenko NM, Sukhoverkhov SV, Semenova TL, Skriptsova AV, Zvyagintseva TN. Seasonal variations of the composition and structural characteristics of polysaccharides from the brown alga *Costaria costata* . Chem Nat Compd. 2009; 45: 786–791.

[4] Imbs TI, Shevchenko NM, Semenova TL, Sukhoverkhov SV, Zvyagintseva TN. Compositional heterogeneity of sulfated polysaccharides synthesized by the brown alga *Costaria costata* . Chem Nat Compd. 2011; 47: 96–97.

[5] Zhang ZY, Chen SK, Chen H. The indoor experiment on rear and culture of *Costaria costata* . Journal of Dalian Fisheries University. 1992; 7: 40–47. (In Chinese)

[6] Moon HJ, Park KS, Ku MJ, Lee MS, Jeong SH, Imbs TI, et al Effect of *Costaria costata* fucoidan on expression of matrix metalloproteinase–1 promoter, mRNA, and protein. J Nat Prod. 2009; 72: 1731–1734. 10.1021/np800797v 19807114

[7] Fu H, Wang KQ, He YH, Ren DD. Functional effect of dietary fiber from seaweed *Costaria costata* residues on reduce in serum lipids in mice. Journal of Dalian Ocean University. 2012; 27: 200–204. (In Chinese)

[8] Saunders GW, Druehl LD. Nucleotide sequences of the small-subunit ribosomal RNA genes from selected Laminariales (Phaeophyta): implications for kelp evolution. J Phycol. 1992; 28: 544–549.

[9] Druehl LD, Mayes C, Tan IH, Saunders GW. Molecular and morphological phylogenies of kelp and associated brown algae. Plant Syst Evol. 1997; 11: 35–221.

[10] Boo SM, Lee WJ, Yoon HS, Kata A, Kawai H. Molecular phylogeny of Laminariales (Phaeophyceae) inferred from small subunit ribosomal DNA sequences. Phycol Res. 1999; 47: 109–114.

[11] Yoon HS, Lee JY, Boo SM, Bhattacharya D. Phylogeny of the Alariaceae, Laminariaceae, and Lessoniaceae (Phaeophyceae) based on plastid-encoded RuBisCo spacer and nuclear-encoded ITS sequences comparisons. Mol Phylogenet Evol. 2001; 21: 231–243. 11697918 10.1006/mpev.2001.1009

[12] Luo XM, Tinker NA, Fan X, Zhang HQ, Sha LN, Kang HY, et al Phylogeny and maternal donor of *Kengyilia* species (Poaceae: Triticeae) based on three cpDNA (*matK*, *rbc*L and *trnH*-*psbA*) sequences. Biochem Syst Ecol. 2012; 44: 61–69.

[13] Smith DR. Mutation rates in plastid genomes: they are lower than you might think. Genome Biol Evol. 2015; 7: 1227–1234. 10.1093/gbe/evv069 25869380 PMC4453064

[14] Clegg MT, Gaut BS, Learn GH, Morton BR. Rates and patterns of chloroplast DNA evolution. P Natl Acad Sci USA. 1994; 91: 6795–6801. 10.1073/pnas.91.15.6795 PMC44285 8041699

[15] Archibald JM. The evolution of algae by secondary and tertiary endosymbiosis. Adv Bot Res. 2012; 64: 87–118.

[16] Doyle JJ, Doyle JL. Isolation of plant DNA from fresh tissue. Focus. 1990; 12: 13–15.

[17] Wang X, Shao Z, Fu W, Yao J, Hu Q, Duan D. Chloroplast genome of one brown seaweed, *Saccharina japonica* (Laminariales, Phaeophyta): its structural features and phylogenetic analyses with other photosynthetic plastids. Mar Genom. 2013; 10: 1–9. 10.1016/j.margen.2012.12.002 23305622

[18] Corguille GL, Pearson G, Valente M, Viegas C, Gschloessl B, Corre E, et al Plastid genomes of two brown algae, *Ectocarpus siliculosus* and *Fucus vesiculosus*: further insights on the evolution of red-algal derived plastids. BMC Evol Biol. 2009; 9: 253 10.1186/1471-2148-9-253 19835607 PMC2765969

[19] Luo R, Liu B, Xie Y, Li Z, Huang W, Yuan J, et al SOAPdenovo2: an empirically improved memory-efficient short-read de novo assembler. GigaScience. 2012; 1: 18 10.1186/2047-217X-1-18 23587118 PMC3626529

[20] Tamura K, Stecher G, Peterson D, Filipski A, Kumar S. MEGA6: Molecular Evolutionary Genetics Analysis version 6.0. Mol Biol Evol. 2013; 30: 2725–2729. 10.1093/molbev/mst197 24132122 PMC3840312

[21] Altschul SF, Madden TL, Schaffer AA, Zhang J, Zhang Z, Miller W, et al Gapped BLAST and PSI-BLAST: a new generation of protein database search programs. Nucleic Acids Res. 1997; 25: 3389–3402. 9254694 10.1093/nar/25.17.3389 PMC146917

[22] Lowe TM, Eddy SR. tRNAscan-SE: a program for improved detection of transfer RNA genes in genomic sequence. Nucleic Acids Res. 1997; 25: 955–964. 9023104 10.1093/nar/25.5.955 PMC146525

[23] Lohse M, Dreshsil O, Bock R. OrganellarGenomeDRAW (OGDRAW): a tool for the easy generation of high-quality custom graphical maps of plastid and mitochondrial genomes. Curr Genet. 2007; 52: 267–264. 17957369 10.1007/s00294-007-0161-y

[24] Kearse M, Moir R, Wilson A, Stones-Havas S, Cheung M, Sturrock S, et al Geneious Basic: an integrated and extendable desktop software platform for the organization and analysis of sequence data. Bioinformatics. 2012; 28: 1647–1649. 10.1093/bioinformatics/bts199 22543367 PMC3371832

[25] Ronquist F, Huelsenbeck JP. MrBayes 3: Bayesian phylogenetic inference under mixed models. Bioinformatics. 2003; 19: 1572–1574. 12912839 10.1093/bioinformatics/btg180

[26] Posada D, Crandall KA. MODELTEST: testing the model of DNA substitution. Bioinformatics. 1998; 14: 817–818. 9918953 10.1093/bioinformatics/14.9.817

[27] Wang L, Mao Y, Kong F, Li G, Ma F, Zhang B, et al Complete sequence and analysis of plastid genomes of two economically important red algae: *Pyropia haitanensis* and *Pyropia yezoensis* . PloS ONE. 2013; 8: e65902 10.1371/journal.pone.0065902 23734264 PMC3667073

[28] Peabody DS. Translation initiation at non-AUG triplets in mammalian cells. J Biol Chem. 1989; 264: 5031–5035. 2538469

[29] Boyen C, Leblanc C, Bonnard G, Grienenberger JM, Kloareg B. Nucleotide sequence of the *cox3* gene from *Chondrus crispus*: evidence that UGA encodes tryptophan and evolutionary implications. Nucleic Acids Res. 1994; 22: 1400–1403. 8190631 10.1093/nar/22.8.1400 PMC307997

[30] Zhang J, Li N, Zhang ZF, Liu T. Structure analysis of the complete mitochondrial genome in cultivation variety ‘Rongfu’. J Ocean Univ China. 2011; 10: 351–356.

[31] Li TY, Qu JQ, Feng YJ, Liu C, Chi S, Liu T. Complete mitochondrial genome of *Undaria pinnatifida* (Alariaceae, Laminariales, Phaeophyceae). Mitochondr DNA. 2014; 1–2. 10.3109/19401736.2013.865172 24409911

[32] Qu JQ, Liu C, Wang XM, Zhang ZB, Liu T. Complete mitochondrial genome of *Costaria costata* shows conservative evolution in Laminariales. Mitochondr DNA. 2014; 1–2. 10.3109/19401736.2013.863290 24409887

[33] Wolfe KH, Li WH, Sharp PM. Rates of nucleotide substitution vary greatly among plant mitochondrial, chloroplast, and nuclear DNAs. P Natl Acad Sci USA. 1987; 84: 9054–9058. 10.1073/pnas.84.24.9054 PMC299690 3480529

[34] Ellegren H. Microsatellite mutations in the germline: implications for evolutionary inference. Trends Genet. 2000; 16: 551–558. 11102705 10.1016/s0168-9525(00)02139-9

[35] Levinson G, Gutman GA. Slipped-strand mispairing: a major mechanism for DNA sequence evolution. Mol Biol Evol. 1987; 4: 203–221. 3328815 10.1093/oxfordjournals.molbev.a040442

[36] Reyes-prieto A, Weber AP, Bhattacharya D. The origin and establishment of the plastid in algae and plants. Annu Rev Genet. 2007; 41: 147–168. 17600460 10.1146/annurev.genet.41.110306.130134

[37] Tajima N, Sato S, Maruyama F, Kurokawa K, Ohta H, Tabata S, et al Analysis of the complete plastid genome of the unicellular red alga *Porphyridium purpureum* . J Plant Res. 2014; 127: 389–397. 10.1007/s10265-014-0627-1 24595640

