# Supplementary material for: Complete Plastid Genome of the Brown Alga Costaria costata (Laminariales, Phaeophyceae)
Source: PLoS One. 2015 Oct 7;10(10):e0140144. doi: 10.1371/journal.pone.0140144 (PMC4596871; doi:10.1371/journal.pone.0140144)
Supplement: S1 Table — (PDF) [file pone.0140144.s002.pdf]

S1 Table. Sequences of the primers designed for gap filling and assembly validation.

Primer Name	Primer Sequence	
	Forward primer (5'-3')	Reverse primer (5'-3')
DL-1	GTACTAGGCAAAGAACTAA	CAACTTATACCTCGACTG
DL-2	TTACGGATCTGAAATGAT	TTATGGCTAAAGCTATGA
DL-3	ATTTCGTTGAGCTTCCTA	TACACTAACAGCCCATCC
DL-4	TTTCGAGGTGAGGGTAAA	ATCCTAATGTTGGTGGGT
DL-5	CGGACGAAAATAATGACG	AGAAACCCCGCACAGAAA
DL-6	GTGGCGGAATTGGCAGAC	AGAAACCCCGCACAGAAA
DL-7	ATTCGCAATCTTGGACAC	TTCATCTTTCGCCACTTT
DL-8	AGAACGTCGAGCAGCAGA	AAGGGATACTTACATCACAGAG
DL-9	TAATTTGTTGACCCGATGC	AAGCCAAGCTCATTTGTTC
DL-10	CAGTTGCCGTAGTTGTGC	TGTCGCTAATTTCGTTCT
DL-11	ATTTAATAACTTTTCGGG	TAAAAGATACCCCAAAAT
DL-12	AAATAGGGTATGGTGGGT	GGCTAGAGGCTGAAGTAA
DL-13	ACATGCTCGTCTTTGAGA	TTAACAGGATCGGGTATT
DL-14	TCGGAAGTTAATTTAGTAAGCG	AAGGCCAGGGACTAGGGT
DL-15	ACTTTACGGCTGTATTGG	AGTTTGTGAGGGTGTCTT
DL-16	TTGTTCTGCGTTGTTGCT	CATCGCGTTAGTGATTGA
DL-17	GGGTTGTTGTTAGTTGCT	TCAGGTAAGTATAAAGGGA
DL-18	TTCCTGTATTACCTCGTAG	TCAAACCCATAGTCTGTTA
DL-19	CTTTTATCGGTAAAGAGG	AACCTACAAGACAAGCAA
DL-20	CGGGAAATTGATTACACC	TGCCCAAGAATGAAGAAC
DL-21	CTGGGTTGGTAAAAGATT	TTACTGAACCAGCCGTAG
DL-22	GGTCTTCCTGGTACATCA	CTCCCAACCGACAAACAT
DL-23	TTATTTGATGCCTTGGTTG	GAGCAGATTTACCTACCG
DL-24	GCTTGCGGTCTTTCTAAC	CTCAAATTACTAGGCATACA
DL-25	GTATGCCTAGTAATTTGAG	CATAACAACGAACAACAC
DL-26	CCGAGGGATTGACAGAGT	CACTTCAACCAGGCACAT
DL-27	AAAGTGAAACTTCGGGTGG	AATGCTTTACGACGAGAC
DL-28	AAGCTATCCCGTCTTCAA	AATGGCTGGGCAAATCTA
DL-29	TAGATTTGCCCAGCCATTT	CTCCTGTTGCCTTCTCCC
DL-30	TAGTTCGGAAATTCCCTT	CTCTTTGAACAACCTCCC
DL-31	GCAGTAATCGGCCATACC	TTCGCTCACCACTACTAACG
