# Supplementary material for: Complete Plastid Genome of the Brown Alga Costaria costata (Laminariales, Phaeophyceae)
Source: PLoS One. 2015 Oct 7;10(10):e0140144. doi: 10.1371/journal.pone.0140144 (PMC4596871; doi:10.1371/journal.pone.0140144)
Supplement: S2 Table — (PDF) [file pone.0140144.s003.pdf]

S2 Table. Substitution rate of 137 shared protein-coding genes among three plastid genomes from Laminariales.

Gene	Length (bp)	Nucleotide Substitution Rate				Amino Acid Substitution Rate			
		Rate	Cc & Up	Cc & Sj	Up & Sj	Rate	Cc & Up	Cc & Sj	Up & Sj
petF	297	20.88%	15.49%	11.78%	18.18%	26.26%	21.21%	12.12%	26.26%
ycf34	288	20.49%	15.28%	0.00%	0.00%	22.92%	17.71%	15.63%	15.63%
ycf40	228	18.42%	13.16%	12.72%	13.60%	23.68%	21.05%	17.11%	13.16%
rps20	306	17.65%	12.09%	9.80%	11.11%	20.59%	15.69%	13.73%	11.76%
ycf41	300	16.33%	13.00%	12.00%	7.67%	25.00%	20.00%	22.00%	12.00%
rpl32	240	15.83%	10.83%	11.25%	10.42%	17.50%	15.00%	10.00%	11.25%
syfB	2304	15.54%	11.72%	10.29%	10.76%	16.80%	12.89%	11.07%	12.24%
rbcR	957	15.26%	10.76%	9.61%	10.66%	12.54%	9.40%	9.09%	7.21%
ilvH	603	14.93%	10.12%	9.95%	11.28%	11.94%	9.45%	7.46%	8.46%
ycf17	171	14.62%	11.11%	7.02%	11.70%	14.04%	8.77%	5.26%	14.04%
rps4	633	14.53%	9.32%	10.58%	10.11%	8.06%	4.27%	7.11%	6.64%
psaE	192	14.06%	11.46%	6.25%	10.94%	14.06%	14.06%	1.56%	14.06%
dnaB	1452	13.43%	9.02%	9.23%	9.30%	9.09%	6.61%	6.40%	6.40%
rps1	798	13.41%	8.65%	8.52%	10.15%	13.53%	9.40%	7.52%	11.28%
rpoC2	3849	13.28%	9.87%	8.18%	9.41%	7.72%	6.70%	4.21%	5.22%
ccs1	1287	12.98%	9.25%	8.31%	9.25%	8.62%	7.46%	4.66%	5.59%
rpl33	240	12.92%	8.75%	3.33%	8.33%	15.00%	8.75%	7.50%	11.25%
ycf33	195	12.82%	11.28%	8.72%	6.67%	12.31%	12.31%	7.69%	6.15%
petJ	339	12.68%	9.44%	6.78%	9.44%	15.93%	13.27%	7.96%	12.39%
ilvB	2172	12.66%	9.12%	8.47%	8.84%	5.52%	4.01%	3.87%	4.14%
ycf37	540	12.22%	7.59%	8.33%	9.26%	6.67%	6.11%	3.33%	4.44%
rpl12	393	12.21%	7.38%	8.65%	9.41%	12.21%	6.87%	8.40%	10.69%
thiG	819	12.09%	8.42%	7.33%	8.79%	8.06%	5.49%	4.40%	6.23%
ycf65	321	11.84%	9.03%	6.85%	8.41%	9.35%	7.48%	4.67%	6.54%
rpl27	255	11.76%	8.24%	6.27%	9.80%	9.41%	7.06%	3.53%	8.24%
chlI	1023	11.73%	9.19%	6.65%	8.50%	1.76%	1.47%	0.29%	1.76%
rps13	381	11.55%	7.61%	6.30%	9.97%	5.51%	4.72%	3.15%	3.94%
dnaK	1893	11.52%	8.40%	7.45%	7.92%	3.33%	2.22%	1.90%	2.69%
rpoA	1158	11.40%	8.55%	7.25%	8.29%	10.36%	6.99%	6.48%	8.55%
rpl20	345	11.30%	8.12%	6.09%	8.99%	3.48%	3.48%	0.00%	3.48%
ycf16	777	11.07%	7.85%	6.56%	8.24%	6.56%	4.25%	3.86%	5.41%
rps3	669	11.06%	7.77%	7.17%	8.37%	2.69%	1.79%	1.35%	2.69%
psaD	399	11.03%	7.02%	6.52%	8.77%	3.01%	2.26%	0.75%	3.01%
secA	2634	10.90%	8.16%	6.99%	7.02%	2.51%	2.16%	1.37%	1.59%
tsf	603	10.78%	6.80%	7.46%	7.63%	4.48%	2.99%	2.99%	2.99%
ccsA	987	10.74%	8.61%	6.99%	6.48%	6.08%	5.47%	3.65%	3.65%
rpoB	3399	10.71%	7.36%	6.80%	7.91%	3.00%	2.03%	1.59%	2.47%
rpl3	591	10.66%	8.12%	5.25%	8.29%	4.57%	2.54%	2.54%	4.06%
acsF	1101	10.63%	7.54%	7.08%	7.18%	4.36%	3.81%	1.36%	3.54%

ycf35	405	10.62%	5.93%	8.15%	6.67%	9.63%	4.44%	5.93%	7.41%
chlN	1359	10.38%	7.43%	6.11%	8.02%	2.87%	2.43%	1.10%	2.43%
groEL	1545	10.36%	7.57%	7.12%	7.06%	2.14%	1.17%	1.17%	2.14%
ycf24	1446	10.03%	6.36%	7.19%	7.26%	2.49%	1.45%	2.07%	1.66%
rpoC1	1833	9.93%	7.15%	5.95%	7.09%	2.29%	1.64%	1.47%	1.64%
clpC	2685	9.87%	7.49%	6.00%	7.11%	1.56%	1.01%	1.01%	1.34%
rpl5	564	9.75%	7.09%	6.38%	6.56%	1.60%	0.53%	1.06%	1.60%
rpl18	330	9.70%	8.18%	6.06%	5.45%	6.36%	4.55%	3.64%	4.55%
rps11	372	9.68%	6.18%	7.26%	6.45%	0.81%	0.81%	0.00%	0.81%
psbV	486	9.67%	7.20%	4.94%	7.82%	4.32%	3.09%	2.47%	4.32%
rpl11	426	9.62%	7.75%	6.57%	5.40%	4.93%	3.52%	3.52%	2.82%
rpl21	312	9.62%	8.01%	4.17%	7.69%	0.96%	0.96%	0.00%	0.96%
rps12	375	9.60%	6.40%	5.87%	7.47%	0.00%	0.00%	0.00%	0.00%
psbW	345	9.57%	6.96%	4.64%	8.12%	0.87%	0.87%	0.00%	0.87%
ycf3	510	9.41%	6.86%	5.88%	6.47%	0.00%	0.00%	0.00%	0.00%
rps16	234	9.40%	5.56%	6.41%	7.26%	1.28%	1.28%	0.00%	1.28%
rps10	321	9.35%	7.48%	4.67%	7.48%	1.87%	1.87%	0.93%	0.93%
ftsH	1887	9.33%	7.00%	5.94%	6.52%	0.95%	0.64%	0.32%	0.95%
ycf42	561	9.27%	6.60%	6.77%	5.88%	3.21%	1.07%	2.67%	2.67%
tatC	783	9.20%	6.64%	5.36%	6.90%	5.36%	3.45%	3.07%	4.21%
cbbx	903	9.19%	6.53%	5.87%	6.42%	0.33%	0.33%	0.33%	0.33%
rps5	504	9.13%	6.35%	5.75%	7.74%	2.98%	1.79%	2.38%	1.79%
secY	1296	9.10%	6.40%	6.17%	6.25%	2.55%	1.85%	1.16%	2.08%
ycf47	231	9.09%	5.63%	6.49%	6.06%	2.60%	1.30%	2.60%	1.30%
petM	99	9.09%	7.07%	3.03%	8.08%	0.00%	0.00%	0.00%	0.00%
rpl19	387	9.04%	6.46%	4.91%	7.24%	5.43%	3.88%	4.65%	2.33%
ycf46	1476	9.01%	6.23%	5.69%	6.37%	0.20%	0.00%	0.20%	0.20%
petA	948	8.86%	6.54%	4.75%	6.75%	5.38%	4.11%	2.85%	4.11%
tufA	1233	8.84%	6.57%	5.11%	6.65%	2.92%	1.70%	1.46%	2.68%
rpl1	693	8.80%	6.35%	5.63%	6.20%	6.06%	5.19%	3.90%	3.46%
chlB	1533	8.74%	6.85%	4.76%	6.52%	1.37%	1.37%	0.59%	0.78%
atpA	1536	8.72%	6.45%	5.21%	6.32%	2.15%	1.56%	1.17%	1.76%
rpl34	138	8.70%	5.07%	5.80%	6.52%	4.35%	0.00%	4.35%	4.35%
rpl16	405	8.64%	6.67%	5.19%	6.17%	1.48%	0.74%	1.48%	0.74%
psaM	93	8.60%	6.45%	6.45%	5.38%	9.68%	6.45%	6.45%	9.68%
ycf4	561	8.56%	6.77%	5.35%	5.17%	2.14%	2.14%	1.60%	0.53%
rps7	471	8.49%	5.73%	4.67%	7.22%	1.27%	0.64%	0.64%	1.27%
rpl2	828	8.45%	5.68%	5.92%	6.04%	2.54%	2.54%	1.45%	1.81%
ycf39	984	8.43%	5.49%	5.89%	6.10%	3.35%	2.13%	1.83%	3.05%
rpl29	192	8.33%	5.21%	5.73%	6.25%	1.56%	0.00%	1.56%	1.56%
rpl6	537	8.19%	6.33%	4.10%	6.15%	6.15%	5.03%	2.79%	4.47%
psaF	609	8.05%	5.25%	4.93%	6.73%	3.94%	2.46%	1.48%	3.94%

atpD	618	7.93%	5.99%	5.18%	5.02%	6.31%	5.83%	2.43%	4.85%
atpG	471	7.86%	5.31%	4.46%	6.16%	3.18%	1.27%	1.91%	3.18%
rps9	429	7.69%	5.13%	5.59%	7.69%	2.80%	1.40%	2.80%	1.40%
petD	483	7.66%	4.97%	5.38%	5.59%	0.00%	0.00%	0.00%	0.00%
rps2	696	7.61%	5.32%	4.74%	5.75%	0.00%	0.00%	0.00%	0.00%
rpl24	252	7.54%	5.56%	5.56%	4.76%	2.38%	0.00%	2.38%	2.38%
atpI	747	7.50%	5.22%	3.88%	6.02%	0.40%	0.40%	0.00%	0.40%
rpl4	648	7.41%	5.56%	4.32%	5.71%	4.63%	3.24%	1.85%	4.17%
chlL	885	7.34%	5.08%	4.18%	5.54%	1.69%	1.69%	1.02%	0.68%
atpB	1446	7.19%	4.77%	4.98%	5.26%	1.66%	1.04%	0.83%	1.66%
rpl22	348	7.18%	2.87%	4.89%	6.90%	2.59%	0.00%	2.59%	2.59%
rpl13	432	7.18%	5.56%	3.94%	5.09%	4.86%	4.86%	2.08%	3.47%
atpE	405	7.16%	5.68%	4.94%	3.95%	3.70%	3.70%	1.48%	2.96%
psbI	126	7.14%	6.35%	5.56%	2.38%	7.14%	7.14%	4.76%	2.38%
psaL	438	7.08%	3.42%	5.48%	5.48%	3.42%	1.37%	2.74%	2.74%
atpF	525	7.05%	5.14%	4.57%	4.95%	3.43%	2.29%	1.71%	2.86%
psbY	114	7.02%	5.26%	3.51%	5.26%	5.26%	5.26%	2.63%	2.63%
rps19	315	6.98%	4.44%	5.71%	3.81%	0.00%	0.00%	0.00%	0.00%
rpl35	159	6.92%	3.77%	3.77%	6.29%	3.08%	1.54%	1.54%	3.08%
rpl31	219	6.85%	5.94%	5.48%	2.74%	2.74%	2.74%	2.74%	0.00%
rpl14	366	6.56%	6.01%	3.01%	4.92%	3.28%	2.46%	1.64%	2.46%
petB	648	6.48%	4.48%	3.86%	5.09%	0.00%	0.00%	0.00%	0.00%
rps18	255	6.27%	4.71%	3.53%	4.31%	1.18%	1.18%	1.18%	0.00%
rps14	303	6.27%	4.62%	4.62%	3.30%	2.97%	2.97%	0.99%	1.98%
ycf19	360	6.11%	5.00%	3.06%	4.17%	2.50%	1.67%	0.83%	2.50%
psaC	246	6.10%	4.88%	3.25%	4.47%	0.00%	0.00%	0.00%	0.00%
psaA	2250	6.09%	4.67%	3.60%	4.22%	0.67%	0.67%	0.00%	0.67%
psaB	2205	5.71%	3.99%	3.85%	3.95%	0.68%	0.41%	0.54%	0.41%
rps8	399	5.51%	3.26%	4.01%	3.76%	0.00%	0.00%	0.00%	0.00%
rbcS	420	5.48%	3.57%	3.33%	4.05%	7.14%	5.71%	3.57%	5.00%
psaJ	129	5.43%	5.43%	2.33%	3.88%	0.00%	0.00%	0.00%	0.00%
rpl9	976	5.33%	3.89%	3.28%	3.69%	4.31%	3.38%	2.46%	2.77%
ycf66	324	5.25%	2.78%	4.32%	3.70%	0.00%	0.00%	0.00%	0.00%
psbC	1425	5.12%	3.93%	3.30%	3.44%	0.42%	0.42%	0.00%	0.42%
psbB	1533	4.70%	3.85%	2.15%	3.65%	0.00%	0.00%	0.00%	0.00%
psbD	1056	4.64%	3.13%	3.03%	3.41%	0.00%	0.00%	0.00%	0.00%
*rbc*L	1467	4.02%	2.25%	2.79%	3.07%	2.45%	1.02%	2.04%	1.84%
atpH	249	3.61%	2.81%	1.61%	2.81%	1.20%	1.20%	0.00%	1.20%
psaI	111	3.60%	1.80%	3.60%	1.80%	0.00%	0.00%	0.00%	0.00%
petG	114	3.51%	2.63%	0.88%	3.51%	0.00%	0.00%	0.00%	0.00%
psbJ	120	3.33%	2.50%	2.50%	2.50%	0.00%	0.00%	0.00%	0.00%
psbX	132	3.03%	0.76%	2.27%	3.03%	6.82%	2.27%	4.55%	6.82%

psbN	132	3.03%	1.52%	2.27%	2.27%	0.00%	0.00%	0.00%	0.00%
rpl23	303	2.97%	2.64%	1.98%	1.32%	1.98%	0.99%	0.99%	1.98%
rpl36	114	2.63%	1.75%	1.75%	1.75%	5.26%	2.63%	5.26%	2.63%
ftrB	348	2.59%	1.72%	2.30%	1.15%	0.86%	0.86%	0.86%	0.86%
rps17	255	2.35%	1.57%	0.78%	2.35%	0.00%	0.00%	0.00%	0.00%
psbL	117	1.71%	0.85%	1.71%	0.85%	0.00%	0.00%	0.00%	0.00%
psbT	126	1.59%	1.59%	0.79%	0.79%	0.00%	0.00%	0.00%	0.00%
psbA	1086	1.57%	1.29%	0.83%	1.01%	0.28%	0.28%	0.00%	0.28%
psbF	132	1.52%	1.52%	0.76%	0.76%	0.00%	0.00%	0.00%	0.00%
ycf12	96	1.04%	1.04%	0.00%	1.04%	0.00%	0.00%	0.00%	0.00%
psbH	204	0.98%	0.49%	0.98%	0.49%	0.00%	0.00%	0.00%	0.00%
psbE	255	0.78%	0.78%	0.39%	0.39%	0.00%	0.00%	0.00%	0.00%
psbK	135	0.74%	0.74%	0.00%	0.74%	0.00%	0.00%	0.00%	0.00%
petN	90	0.00%	0.00%	0.00%	0.00%	0.00%	0.00%	0.00%	0.00%
		9.55%	6.87%	5.97%	6.79	5.57%	4.49%	3.83%	3.35%
