# Supplementary material for: Complete Plastid Genome of the Brown Alga Costaria costata (Laminariales, Phaeophyceae)
Source: PLoS One. 2015 Oct 7;10(10):e0140144. doi: 10.1371/journal.pone.0140144 (PMC4596871; doi:10.1371/journal.pone.0140144)
Supplement: S3 Table — (PDF) [file pone.0140144.s004.pdf]

S3 Table. Substitution rate of 35 shared protein-coding genes among three mitochondrial genomes from Laminariales.

Gene	Length (bp)	Substitution Rate	*Sj* & *Up*	*Sj* & *Cc*	*Up* & *Cc*
rps11	571	28.20%	21.89%	16.29%	17.69%
rpl31	221	26.24%	15.84%	19.91%	18.55%
rpl2	765	25.75%	21.57%	15.56%	16.99%
rpl16	415	24.82%	16.87%	18.07%	16.39%
tatC	780	22.82%	18.21%	8.59%	19.23%
cox2	4083	22.80%	16.97%	15.33%	14.99%
rps3	870	22.30%	15.40%	14.14%	16.78%
rpl5	560	21.79%	16.25%	13.21%	16.25%
rps4	816	21.45%	14.95%	14.46%	14.95%
rps10	351	21.37%	14.53%	13.96%	14.53%
rps2	669	21.08%	13.60%	15.10%	15.99%
rpl14	387	20.93%	14.21%	14.99%	14.47%
rps13	360	20.56%	13.61%	15.00%	13.61%
rpl6	492	19.51%	14.84%	11.99%	13.01%
rps12	387	18.86%	14.73%	13.18%	11.63%
rps7	692	18.79%	12.86%	13.87%	11.27%
nad11	632	18.67%	13.45%	11.55%	13.92%
nad6	947	18.37%	14.57%	11.72%	12.14%
rps14	297	16.84%	12.12%	10.10%	13.13%
nad5	1989	16.79%	11.81%	10.76%	12.57%
nad2	1497	16.57%	12.49%	11.42%	10.55%
atp6	753	15.67%	14.61%	3.32%	13.81%
nad7	1197	15.46%	13.28%	5.93%	12.36%
cox3	820	15.24%	11.10%	9.88%	10.98%
cob	1257	15.12%	10.10%	10.74%	10.50%
cox1	1605	15.02%	10.72%	10.59%	10.03%
nad4	1452	14.74%	10.67%	9.64%	10.81%
nad3	363	14.60%	10.19%	10.47%	10.19%
nad1	981	13.86%	9.68%	9.58%	9.17%
nad9	588	13.78%	9.52%	9.35%	9.69%
nad4L	303	12.87%	9.57%	7.92%	8.58%
rps8	738	10.16%	6.78%	7.45%	6.78%
rps19	270	10.00%	6.30%	7.04%	7.41%
atp8	261	7.66%	5.36%	6.13%	4.98%
atp9	228	7.46%	3.95%	5.70%	5.26%
			13.44%	11.69%	12.75%
